# Barriers and enablers to detection and management of chronic kidney disease in primary healthcare: a systematic review

**DOI:** 10.1186/s12882-020-01731-x

**Published:** 2020-03-12

**Authors:** Elizabeth P. Neale, Justin Middleton, Kelly Lambert

**Affiliations:** 1 School of Medicine, Faculty of Science, Medicine and Health, University of Wollongong, Wollongong, NSW 2522 Australia; 2 Illawarra Health and Medical Research Institute, University of Wollongong, Wollongong, NSW 2522 Australia; 3 Health Impacts Research Cluster, University of Wollongong, Wollongong, NSW 2522 Australia; 4 Department of Clinical Nutrition, Wollongong Hospital, Level 5, Block C, Crown St, Wollongong, NSW 2500 Australia

**Keywords:** Chronic kidney disease, Primary care, Barriers, Enablers, Systematic review

## Abstract

**Background:**

Chronic kidney disease (CKD) is growing population health concern worldwide, and with early identification and effective management, kidney disease progression can be slowed or prevented. Most patients with risk factors for chronic kidney disease are treated within primary healthcare. Therefore, it is important to understand how best to support primary care providers (PC-P) to detect and manage chronic kidney disease. The aim of this systematic review was to evaluate barriers and enablers to the diagnosis and management of CKD in primary care.

**Methods:**

A systematic review of qualitative research on the barriers and/or enablers to detection and/or management of CKD in adults within primary healthcare was conducted. The databases Medline (EBSCO), PubMed, Cochrane CENTRAL, CINAHL (EBSCO) and Joanna Briggs Institute Evidence Based Practice (Ovid) were searched until 27th August 2019. Barriers and/or enablers reported in each study were identified, classified into themes, and categorised according to the Theoretical Domains Framework.

**Results:**

A total of 20 studies were included in this review. The most commonly reported barriers related to detection and management of CKD in primary care were categorised into the ‘Environmental context and resources’ domain (*n* = 16 studies). Overall, the most common barrier identified was a lack of time (*n* = 13 studies), followed by a fear of delivering a diagnosis of CKD, and dissatisfaction with CKD guidelines (both *n* = 10 studies). Overall, the most common enabler identified was the presence of supportive technology to identify and manage CKD (*n* = 7 studies), followed by the presence of a collaborative relationship between members of the healthcare team (*n* = 5 studies).

**Conclusion:**

This systematic review identified a number of barriers and enablers which PC-P face when identifying and managing CKD. The findings of this review suggest a need for time-efficient strategies that promote collaboration between members of the healthcare team, and practice guidelines which consider the frequently co-morbid nature of CKD. Enhanced collaboration between PC-P and nephrology services may also support PC-Ps when diagnosing CKD in primary care, and facilitate improved patient self-management.

## Background

Chronic kidney disease (CKD) is growing population health concern worldwide. The results of the Global Burden of Disease Study suggested that in 2015, 1.2 million deaths were associated with kidney failure, an increase of 32% since 2005 [1]. Higher income countries typically spend 2–3% of their annual health budget on the treatment of end-stage kidney disease, however the percentage of the population which receives such treatment is less than 0.03% of the population [2]. Even a moderate decline in kidney function is associated with significantly higher risk of cardiovascular events and mortality, while those who progress to end-stage kidney disease require specialist treatment, either transplantation, dialysis or palliation, all adding significant cost to the health budget [3].

With early identification and effective management, CKD progression to end-stage kidney disease can be slowed or prevented. Most patients with risk factors for, or early stages of CKD are treated in primary care [4–6]. Therefore, exploration of how best to support primary care providers (PC-P) to detect and manage CKD is needed. While several studies have explored factors impacting the management of CKD in the primary care setting [7–10], there is a need to identify common barriers and enablers in order to develop effective strategies to enhance CKD care. While a systematic review of barriers to CKD management in primary care has been published [11], this study did not explore enabling factors. In addition, as the search for the previous review was conducted almost 10 years ago, there is a need to explore the more recent evidence on this topic. Therefore, the present systematic review aimed to provide an expanded and more recent perspective on the topic of barriers and enablers to the diagnosis and management of CKD in primary care.

## Methods

This systematic review is reported according to the Preferred Reporting Items for Systematic Reviews and Meta-Analyses statement [12]. The review protocol was registered with the International Prospective Register of Systematic Reviews (CRD42018092364, http://www.crd.york.ac.uk/).

Systematic searches were performed using the Medline (EBSCO), PubMed, Cochrane CENTRAL, CINAHL (EBSCO) and Joanna Briggs Institute Evidence Based Practice (Ovid) databases to identify relevant articles (Supplementary Material 1). After initially being conducted in April 2018, the search was then updated on 27th August 2019. Studies which reported qualitative information on the barriers and/or enablers to detection and/or management of CKD (stages 1–5) in adults (over 18 years) within primary healthcare were eligible for inclusion in the review. Identification of primary healthcare settings was based on The Department of Health Australian Government [13] and Australian Institute of Health and Welfare [14] definitions of primary healthcare. Studies were limited to those which reported qualitative information in order to facilitate in-depth analysis of the reported barriers and enablers, in line with a previous systematic review conducted in CKD [15]. Studies were excluded in the case they: reported barriers or enablers in both primary and secondary/tertiary healthcare where it was not possible to differentiate the two settings; where the study was a review/study protocol/case study; or where the study was undertaken in a developing country, in order to ensure findings were comparable across health systems. Eligible studies were also limited to those published in English.

Retrieved studies were screened by title and abstract using the semi-automated citation screening tool Abstrackr [16]. Full texts were retrieved for potentially relevant articles and reviewed against the inclusion/exclusion criteria. Contentious articles were discussed with another researcher (EN or KL) until consensus was reached.

Study context, data collection method, country, participant characteristics and sample size were extracted into summary tables. All included studies were assessed for their methodological quality using the Joanna Briggs Institute Critical Appraisal Checklist for Qualitative Research [17].

Barriers and/or enablers reported in each study were identified. Similar barriers/enablers were then grouped into themes [15], and categorised according to the Theoretical Domains Framework [18]. The Theoretical Domains Framework consists of 12 theoretical domains related to behaviour change [19]. These domains can be used to map potential areas to target for implementation challenges [20, 21]. In addition to the published domains, we created an additional domain (*perceptions of patients*) to fully encapsulate all barriers and enablers observed in the current study. Exemplar quotes were then identified for each theme.

## Results

### Characteristics of included studies

**Fig. 1:**
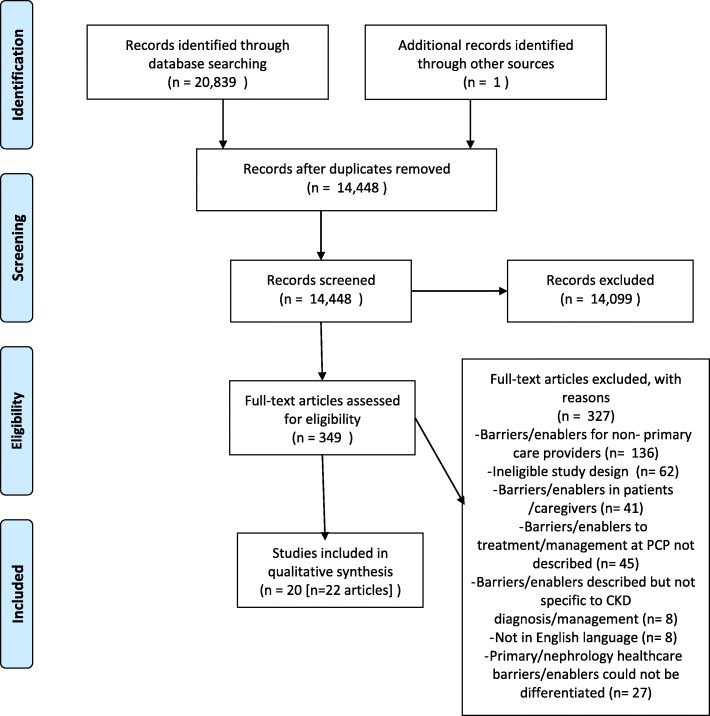
PRISMA flow diagram of study selection

Across the original and updated searches, a total of 20,840 results were obtained (Fig. 1). After removal of duplicates, 14,448 results were screened by title and abstract using the citation screening program Abstrackr [16]. A total of 349 potentially relevant articles were retrieved for full text review, with 22 articles describing 20 studies included in the review.

Of the 20 included studies, 12 were interviews [7, 10, 22–31], six were focus groups [5, 6, 8, 32–36], and two were surveys with open-ended responses [9, 37] (Table 1). General practitioners, nurses, practice managers, pharmacists and medical assistants were represented across the 20 studies. Included studies were performed in the United Kingdom [6, 7, 10, 22, 32], United States [8, 23–26, 28, 29, 33, 34], Australia [5, 9, 30, 36], Canada [27, 31, 37], and the Netherlands [35].

**Table 1:** Characteristics of included studies

Citation	Context	Data collection method	Country	Participant characteristics	Sample size
Armstrong et al., 2016 [22]	Observations, interviews and documentary analysis of the ENABLE-CKD project, which aimed to close the gap between guidelines and practice	Interviews	United Kingdom	Primary care staff across general practices (general practitioners, nurses, practice managers, pharmacist, self-management facilitator, administrator/ support staff)	24 (general practitioners: *n* = 7, nurses: *n* = 9, practice managers: m = 4, pharmacist: *n* = 1)
Blakeman et al. 2012 [7]	Exploration of CKD management in primary care, within practices participating in renal collaborative project	Interviews	United Kingdom	General practitioners and nurses	21 (general practitioners: *n* = 11, nurses: *n* = 10)
Crinson et al. 2010 [32]	Exploration of primary care practitioners views of CKD and its management	Focus groups	United Kingdom	General practitioners and practice nurses	36 (*n* = 26 general practitioners, *n* = 9 practice nurses, n = 1 practice-based pharmacist)
Danforth et al. 2019 [29]	Identification of risk factors, facilitators, and barriers to follow-up of abnormal eGFR results for diagnosing CKD	Interviews	United States of America	Primary care physicians	15
Gheewala et al. 2018 [30]	Exploration of community pharmacists barriers to implementing a CKD risk assessment service	Interviews	Australia	Community pharmacists	8
Greer et al. 2012 [33]	Exploration of primary care providers’ barriers to educating patients about CKD	Focus groups	United States of America	Primary care providers (physicians and nurse practitioners)	18 (*n* = 15 physicians, n = 3 nurse practitioners)
Greer et al. 2015 [23]	Exploration of barriers to preparing patients for renal replacement therapy	Interviews	United States of America	Primary care physicians	4^a^
Greer et al. 2019 [8], Sperati et al. 2019 [34]	Exploration of primary care physicians’ perceived barriers and facilitators to management of CKD in a) primary care, and b) at the primary care-nephrology interface	Focus groups	United States of America	Primary care physicians	32
Litvin et al. 2016 [24]	Exploration of whether clinical decision support could be used to improve identification and management of CKD	Group interviews	United States of America	Medical doctors, licensed practical nurse, nurse practitioner, registered nurse, medical assistant, physician assistant	11 practices (ranging in size from 1 to 8 providers)
Lo et al. 2016 [5] and Lo et al. 2016 [36]	Exploration of factors influencing health care of diabetes and CKD	Focus groups	Australia	General practitioners	22^a^
McBride et al. 2014 [25]	Exploration of primary care providers’ attitudes regarding a CKD registry and its implementation	Interviews	United States of America	Primary care providers (physicians, nurse practitioners)	20 (*n* = 19 physicians, *n* = 1 nurse practitioner)
Nash et al. 2018 [31]	Exploration of primary care providers’ perceptions of barriers and enablers to following guidelines for requesting creatinine tests to confirm CKD	Interviews	Canada	Primary care providers (physicians and nurse practitioners)	13 (*n* = 9 physicians, *n* = 4 nurse practitioners)
Nihat et al. 2016 [6]	Process evaluation of the Quality Improvement in CKD study, which compared audit-based education and sending clinical guidelines and prompts with usual care	Focus groups	United Kingdom	General practice (including general practitioner, practice nurses, healthcare assistants and practice manager)	4 practices (including 6–9 members of the multi-professional team in each group)
Sinclair et al. 2017 [9]	Identification of barriers and facilitators to CKD screening practices in practice nurses	Cross-sectional survey (open-ended questions)	Australia	Practice nurses	26
Smith et al. 2012 [26]	Analysis following change to automatic reporting of eGFR in all laboratory results (previously only serum creatinine reported)	Interviews	United States of America	Primary care providers (physicians, nurse practitioners, physician assistants)	19 (*n* = 13 physicians, *n* = 2 nurse practitioners, *n* = 4 physician assistants
Tam-Tham et al. 2016 [27]	Description of primary care physicians perceptions of key barriers, facilitators, and strategies to enhance conservative care for community-dwelling older adults with Stage 5	Interviews	Canada	Primary care physicians	27
Tam-Tham et al. 2016 [37]	Examination of perceived barriers, facilitators to improve primary care physicians’ ability to conservatively manage older adults with Stage 5 who were not planning to initiate dialysis	Cross-sectional survey^b^	Canada	Primary care physicians	409
Tonkin-Crine et al. 2015 [10]	Exploration of general practitioners views and experiences of managing patients with advanced CKD and referral to secondary care	Interviews	United Kingdom	General practitioners	19
van Dipten et al. 2018 [35]	Exploration of perspectives of general practitioners familiar with CKD management guidelines, including the applicability of national interdisciplinary guidelines	Focus groups	The Netherlands	General practitioners	27
Vest et al. 2015 [28]	Process evaluation of TRANSLATE-CKD study, a randomised controlled trial examining implementation of evidence-based CKD guidelines in primary care practice. Interviews conducted at baseline to assess current practice	Interviews	United States of America	Primary care clinicians	27 (*n* = 24 doctors, *n* = 3 nurse practitioners/physician assistants) interviewed

^a^Plus additional secondary or tertiary care practitioners who were not included in the present review

^b^Open-ended responses only included in this review

The methodological quality according to the Joanna Briggs Institute critical appraisal checklist is summarised in Table 2. All studies were assessed as having congruity between the stated philosophical perspective and the research methodology used; the research methodology and research questions; the research methodology and methods used to collect data; and the methodology and the representation and analysis of data. A statement locating the researcher culturally/theoretically was reported in four studies and the influence of the researcher on the research was reported in two studies. Participant voices were adequately represented in all studies, meaning that study conclusions were demonstrated to be based on results, for example quotes from participants. Adequate conclusions were drawn from the analysis/interpretation of the data in all studies. All studies provided a statement of ethical approval.

**Table 2:** Assessment of methodological quality of included studies

Citation	Q1	Q2	Q3	Q4	Q5	Q6	Q7	Q8	Q9	Q10
Armstrong et al. 2016 [22]	Y	Y	Y	Y	Y	N	N	Y	Y	Y
Blakeman et al. 2012 [7]	Y	Y	Y	Y	Y	N	N	Y	Y	Y
Crinson et al. 2010 [32]	Y	Y	Y	Y	Y	N	N	Y	Y	Y
Danforth et al. 2019 [29]	Y	Y	Y	Y	Y	N	N	Y	Y	Y
Gheewala et al. 2018 [30]	Y	Y	Y	Y	Y	N	N	Y	Y	Y
Greer et al. 2012 [33]	Y	Y	Y	Y	Y	N	N	Y	Y	Y
Greer et al. 2015 [23]	Y	Y	Y	Y	Y	N	N	Y	Y	Y
Greer et al. 2019 [8], Sperati et al. 2019 [34]	Y	Y	Y	Y	Y	Y	Y	Y	Y	Y
Litvin et al. 2016 [24]	Y	Y	Y	Y	Y	N	N	Y	Y	Y
Lo et al. 2016a [5]/Lo et al. 2016b [36]	Y	Y	Y	Y	Y	Y	Y	Y	Y	Y
McBride et al. 2014 [25]	Y	Y	Y	Y	Y	N	N	Y	Y	Y
Nash et al. 2018 [31]	Y	Y	Y	Y	Y	N	N	Y	Y	Y
Nihat et al. 2016 [6]	Y	Y	Y	Y	Y	N	N	Y	Y	Y
Sinclair et al. 2017 [9]	Y	Y	Y	Y	Y	N^a^	N^a^	Y	Y	Y
Smith et al. 2012 [26]	Y	Y	Y	Y	Y	N	N	Y	Y	Y
Tam-Tham et al. 2016a [27]	Y	Y	Y	Y	Y	N	N	Y	Y	Y
Tam-Tham et al. 2016b [37]	Y	Y	Y	Y	Y	N^a^	N^a^	Y	Y	Y
Tonkin-Crine et al. 2015 [10]	Y	Y	Y	Y	Y	N	N	Y	Y	Y
Van Dipten et al. 2018 [35]	Y	Y	Y	Y	Y	Y	N	Y	Y^b^	Y
Vest et al. 2015 [28]	Y	Y	Y	Y	Y	Y	N	Y	Y	Y

*Y* Yes, *N* No

^a^Note this study methodology would mean minimal bias could be given from the researcher

^b^Study provides statement that ethical approval was not required

### Barriers to identification and management of CKD in primary care

The barriers to detection and management of CKD in primary care identified in this review could be categorised into seven domains of the Theoretical Domains Framework, outlined in detail below. The domains and corresponding themes are shown by study in Table 3, and exemplar quotes for each theme are listed in Table 4.

**Table 3:** Barriers to diagnosis and management of CKD in primary care, as reported in included studies (studies listed by reference number)

	[22]	[7]	[32]	[29]	[30]	[23]	[8, 34]	[33]	[24]	[5, 36]	[25]	[31]	[6]	[9]	[26]	[27]	[37]	[10]	[35]	[28]
Beliefs about capabilities																				
Challenges educating patients								X											X	
Challenging nature of CKD management							X									X		X		
Beliefs about consequences																				
Cost and/or burden for patients				X			X													
Fear of frightening patients with diagnosis	X	X	X					X	X				X	X	X			X	X	
Lower priority of CKD as a clinical issue	X	X						X				X		X					X	
Perception that kidney decline is to be expected in aging		X	X										X						X	
Reactive focus to healthcare										X										
Environmental context and resources																				
Challenges using laboratory measures for CKD diagnosis or management			X				X					X								
Inadequacy of reporting process to support quality improvement																				X
Lack of patient education resources							X	X									X			
Lack of renumeration for CKD	X													X						
Limited access to nephrology							X			X							X		X	
Technological issues				X					X											X
Time/workload	X		X	X	X		X	X				X	X	X	X	X			X	X
Variation in practice style	X																			
Knowledge																				
Dissatisfaction with guidelines			X				X		X		X	X	X			X		X	X	X
Lack of awareness of guidelines			X	X			X													X
Lack of awareness of resources/support services																X	X			
Perceived lack of adequate knowledge or training			X				X							X		X	X	X	X	X
Perceived lack of clear definition of CKD																			X	
Perceptions about patients																				
Lack of patient understanding of CKD					X		X	X										X		
Perceived low patient adherence			X		X					X		X								X
Social influences																				
Poor communication between healthcare providers					X		X			X						X	X		X	
Social/professional role and identity																				
Lack of clear role delineation between healthcare providers		X		X			X			X		X				X				
Perception of role by other healthcare providers	X					X	X			X				X					X	
Patient perception of roles of healthcare provider/s					X		X							X

**Table 4:** Exemplar quotes illustrating barriers to diagnosis and management of CKD in primary care, by theme^a^

Domain/theme	Quote
Beliefs about capabilities	
Challenges educating patients	“I think the kidney is very complex…and I think patients have a hard time grasping kidney disease because they don’t feel it at all, they just don’t…. When you start talking pathophys to patients who are mostly, in my patient population, working class, blue collar, a lot of them have not finished high school, you just need to keep things very simple and I don’t think the kidney is simple.” [33]
Challenging nature of CKD management	“If you are a young person with [CKD] four and five it’s much more clear cut as to what you are treating and how you manage it compared to an elderly person when there is all this comorbidity, you know, they have all got diabetes, they have all got ischaemic heart disease, very few of them have just got renal disease. The care is much more complicated.” [10]
Challenging nature of CKD management	“If the blood pressure is high, I put them on blood pressure medicine, and I fixed it. If you have chronic kidney disease, you still have chronic kidney disease. You can’t fix it. All you can do is [ensure].. . it doesn’t worsen. We’re not helping…it’s not very exciting.” [8, 34]
Beliefs about consequences	
Cost and/or burden for patients	“Somebody’s taken a day off of work to bring mom in who has otherwise no transport, so that person’s already out of work. Do you think they want to take another vacation day to come back in two weeks? No.” [8, 34]
Fear of frightening patients with diagnosis	‘So, I try not to panic them ... they don’t like this CKD label, which is why I don’t tend to dwell on that, perhaps, very much, I tend to just skim over it and then go into the explanation rather than saying each time they come, “oh yes, and you’ve got CKD, haven’t you?” [7] “It’s like other things, if you use the word “kidney failure” or “heart failure” people instantly think “oh my goodness, I’m going to drop dead tomorrow”.” [10] “When I have had these consultations with patients, their face changes. You almost feel like you have kind of upset them, and it took a lot of my own energy and training to capture it in that consultation, bring them back and sell it to them to say, “This is no reason for panic”, but it always sounded hollow because they still remained anxious for quite a while. And I felt, when I spoke to the other GPs, perhaps that is why they kind of kept delegating it to different people rather than take ownership themselves, whereas they were much more comfortable selling IHD and diabetes.” [7]
Lower priority of CKD as a clinical issue	“So I’ll tell you what, we have 49 diseases that we deal with in family medicine. Kidneys are one small one, and there’s very little to do with that repeat creatinine. There’s nothing that changes. So is it a priority? No. There are many other things that are higher priority.” [31] “I had somebody sitting in that chair yesterday—I was more concerned about their liver and he said “oh, how are the kidneys?” and they were fine, he’s got really good EGFR. He could live out his life without any problems but he’s now spending every day worrying about his kidneys. It’s medicalising something in the patient’s mind and exaggerating the impact of it on their lives.” [22]
Perception that kidney decline is to be expected in aging	“I mean I think that’s the issue, because I suppose CKD in an eighty year old, you’ve got an eGFR of 59 (ml/min/1.73 m2), is that really CKD or is that just you are 89. I think certainly where I would hope the others have discussed, certainly I am, is ... if you’ve got CKD or you’re young and you’ve got proteinuria, definitely that is a really important thing to hammer in. But yeah, 80/90 year olds, I wouldn’t suggest we’re probably discussing it, if they’ve got a mild CKD3.” [7]
Reactive focus to healthcare	“Until we focus on prevention and making people leaner, we’re not going to succeed” [5, 36]
Environmental context and resources	
Challenges using laboratory measures for CKD diagnosis or management	“The lab did not calculate the GFR.. .I think that we probably missed a lot.. . [because] a creatinine 1.3.. .looks all right.. ..” [8, 34]
Lack of patient education resources	“There’s no kidney educator to send them to.” [33]
Lack of renumeration for CKD	“Screening activity for any chronic disease is not Medicare rebatable so therefore not economical use of nursing time” [9]
Limited access to nephrology	“Consultant appointments are too far out and unavailable when I need them.” [37]
Technological issues	“I have patients that have truly had CKD 3 for 2 or 3 years, but nobody has really talked to them…I understand how that can be because it shows up as a normal lab…and I kind of feel like maybe somebody who has a GFR less than 60 who has CKD 3, even though their creatinine is in the normal range, maybe that shouldn’t just show up as a normal lab. Because when we’re so busy and you’re really quickly going through…sometimes people don’t see numbers; they see colors…if there’s no color coding, nothing that says there’s anything abnormal in this result, they may not even look at the results. They say okay, the computer is telling me it’s normal…” [29]
Time/workload	“I think during the 15 or 20 min you have with the patient appointment, your agenda’s long. You need to deal with their blood pressure and their diabetes and they may come in because their back’s hurting or something else.” [8, 34] “Labs sometimes will be a little difficult because…that’s too many people to keep track of, but that’s how many more results that come into your basket. So then if you’re busy in clinic and then you’re busy managing, juggling some other things throughout the day, you probably won’t get to it till the evening, and sometimes you’re very tired.” [29] “I would say the challenge is they’re patients who have numerous comorbidities. There are time challenges for us with a busy office. They are patients who take a lot of time. They often are on numerous medications, they require a lot of blood work for monitoring, and they often have a high rate of hospital admissions for whether it’s their renal problem or it’s the diabetes, or there’s congestive heart failure, or pneumonia.” [27]
Knowledge	
Dissatisfaction with guidelines	“And I think because a lot of those guidelines and rules change over time, there’s just a lot of confusion. So I think it is kind of this squishy black hole to a lot of primary care doctors as far as the nitty gritty details.” [8, 34] “I’m going to assume that [guidelines] are evidence based or at least partially evidence based as much as guidelines can be because if you look at those guidelines in general they’re about maximally 14% evidence based and the rest is opinion, so I assume that they are approximately the same as every other guideline.” [31]
Lack of awareness of guidelines	“I know there’s like the National Kidney Foundation, but I feel like the ADA guidelines are much more useful.. . I mean I certainly don’t know them [CKD guidelines] very well and I can’t visualize an algorithm from them.” [8, 34]
Lack of awareness of resources/support services	“Did not know conservative clinic existed. Need to promote the palliative nephrology clinic.” [37]
Perceived lack of adequate knowledge or training	“[there is a] barrier just because of my limited knowledge/experience.” [37] “I feel like there’s a lot of areas within medicine that I know a lot about.. .but renal.. ..It’s not my super comfort zone” [8, 34]
Perceived lack of clear definition of CKD	“The initial question was what is your picture of chronic kidney damage, and honestly, that picture is just a check mark in a row of risk factors.” [35]
Perceptions about patients	
Lack of patient understanding of CKD	“[Patients] don’t understand what [CKD] actually means. Especially those who don’t really have symptoms, there are lots of people with CKD 5 that don’t have symptoms ... it’s “life’s all fine, how can my kidneys be failing? I feel fine” ... I think because they don’t have symptoms, often they don’t really understand the importance of it.” [10]
Perceived low patient adherence	“It’s a willingness to change, it’s often diet and smoking related, so you’ve got the numbers and you try and work against the numbers, but you know in your heart that unless you put every single medication in the book into that person, and you’re not going to, you’re not going to hit the targets.” [32] “getting the patients to care as much as I do.” [28]
Social influences	
Poor communication between healthcare providers	“The disappointing thing was that once I made that phone call [to the nephrologist], I never got any documentation or phone calls back from that service, and I had to find out by reading in the newspaper that she had died.” [27] “Unfortunately, there’s a pretty big disconnect between primary practice and tertiary. There still is. There probably always will be because – there are some units which are very good at communicating with me and try quite earnestly to keep in contact, but other ones who don’t” [5, 36]. “…some of the medications that the nephrologists use I don’t use. I mean I don’t start [the patient] on it, but when they refer back I don’t know how long I’m supposed to keep them on the medications or is it safe. The last thing that as a primary care physician I want to do is hurt my patient. By not knowing that oh, you shouldn’t have kept them on that, well I didn’t know that. You didn’t tell me. There’s no note.” [8, 34]
Social/professional role and identity	
Lack of clear role delineation between healthcare providers	“And so then the [part time specialist in urgent care or the ED]…they say well, I’m only here once a week so I’ll just cc it to the primary and the primary will deal with it. And the primary says hey, I didn’t order this lab. I don’t own the lab… so whoever ordered it…I’m assuming is going to manage this and take care of it and…let this patient know. So, there’s that. I think that’s probably one of our bigger gaps.” [29] “Often I’ll send them in with all their blood tests and they’ll immediately do another set at the hospital” [5, 36]. “And I don’t feel like the nephrologists do a very good job of like sending [a consult note]- - to me to say I’m following her, you’re following her, is somebody following her.” [8, 34]
Perception of role by other healthcare providers	“some general practitioners do not believe the nurse should be screening or consulting with patients as they believe that it is their role, not the nurses” [9] “And you do get judged by your lowest common denominator (…) you only need one or two bad stories and then that sets a reputation within the system that ‘We don’t trust GPs’ or ‘GPs don’t do this well’” [5, 36]. “…they just don’t get the relationship. They really don’t understand it…you guys don’t even say thank you. I’m referring my patient to you. You do not give me the third degree or say what I have to do….if we’re going to jump through hoops [for you] to see my patient then okay, I’ll send my patient somewhere else. You can’t do that in private practice so the nephrologist or any specialist is not going to do that. They’re going to send a note, they’re going to say thank you for sending your very lovely [patient]...” [8, 34] “…but I can tell you that a lot of times even though I’m extremely well-trained, [to the nephrologist] I’m [the] stupid primary care doctor who doesn’t seem to know anything…” [8, 34]
Patient perception of roles of healthcare provider/s	“And a lot of patients will just ignore what the specialist says because they trust their primary care doctor, and so you find out…six months later that they were supposed to be taking something different as far as the nephrologist was concerned” [8, 34] “some patients believe it is their doctor’s role to discuss their health concerns, rather than the nurse who is only there to perform basic care” [9] “…they [patients] also spent $60 and they’re like why don’t you just do that? He [the nephrologist] didn’t do anything that you didn’t do” [8, 34]

^a^In addition to the themes listed in the table, the following themes were identified in the primary studies without quotes provided: *inadequacy of reporting process to support quality improvement; variation in practice style*

#### Environmental context and resources

The most commonly reported barriers related to detection and management of CKD in primary care were categorised into the ‘Environmental context and resources’ domain and were reported in 16 studies [5, 6, 8, 9, 22, 24, 26–37]. PC-Ps frequently perceived they lacked time to devote to this task [6, 8, 9, 22, 26–35] and this was exacerbated by the limited amount of time available for patient appointments. The complex nature of managing multiple co-morbidities also meant additional time was needed for these patients. Limited access to specialist nephrology services was also highlighted as a barrier [5, 8, 34–37], and was perceived to result in delays in patients being able to make appointments. Other factors included challenges interpreting laboratory measures [8, 31, 32, 34], for example a study conducted in the United States highlighted a barrier of laboratory results providing creatinine but not calculating eGFR [34]. A lack of educational resources for patients [8, 33, 34, 37] was reported, although minimal detail was provided on the type of resources (for example brochures, online materials) that were desired. In addition, technological issues such as software not automatically flagging abnormal results [24, 28, 29]; and a lack of renumeration for tasks such as CKD screening within health system rebates [9, 22] were reported. Attempts to implement changes in the primary care setting were obstructed by variations in practice operations [22], as well as a lack of CKD-specific information in insurance reports and other performance data [28].

#### Beliefs about consequences

A total of 14 studies reported barriers related to ‘beliefs about consequences’ [5–10, 22, 24, 26, 29, 31–36]. The most commonly reported barrier within this domain was a fear of frightening patients by delivering a diagnosis of CKD. This was reported in 10 studies [6, 7, 9, 10, 22, 24, 26, 32, 33, 35]. The source of fear for practitioners in primary care appeared to frequently be due to the perception that patients did not understand CKD and therefore would not be able to cope with the diagnosis. Some studies also reported a perception that CKD was a lower priority clinical issue [7, 9, 22, 31, 33, 35], particularly in light of other co-morbidities which also required management. There was also a reported perception that declining kidney function was an expected part of aging [6, 7, 32, 35], and therefore it was more important to focus on overall quality of life rather than CKD. An additional barrier identified within this domain was the perceived cost to patients of managing CKDs [8, 29, 34], particularly the time and financial burden associated with attending multiple appointments and multiple blood tests. One study also reported that the current approach to healthcare was too reactive [5, 36], and did not prioritise preventative measures that might minimise the risk of developing CKD, such as weight management.

#### Knowledge

Thirteen studies reported barriers to the identification and management of CKD in the ‘knowledge’ domain [6, 8–10, 24, 25, 27–29, 31, 32, 34, 35, 37]. A commonly reported barrier was a dissatisfaction with the current evidence based guidelines for the management of CKD [6, 8, 10, 24, 25, 27, 28, 31, 32, 34, 35]. The theme of dissatisfaction with guidelines covered a range of concepts including where participants found guidelines confusing, or felt that they were not appropriate. Numerous studies reported that those in primary care felt the guidelines were confusing, difficult to use, or changed frequently. It was also identified that there was a perception that there is inadequate training or education for PC-P on the management of CKD [8–10, 27, 28, 32, 34, 35, 37], resulting in some practitioners feeling unprepared to diagnose or manage patients with CKD. Other studies reported that practitioners were unfamiliar with management guidelines for CKD [8, 28, 29, 32, 34] (categorised into the theme ‘lack of awareness of guidelines’), as well as available resources or support services, for example for conservative care [27, 37]. Finally, there was a perception that the definition of CKD was not clear and resulted in diagnostic challenges [35].

#### Social/professional role and identity

A number of barriers relating to the professional role of PC-Ps were reported. Six studies [5, 8, 9, 22, 23, 34–36] reported barriers that were related perceived misunderstanding about the role of PC-Ps in the management of CKD by other healthcare professionals (for example nephrologists). Some studies reported that general practitioners felt that other healthcare providers underestimated their role [5, 8, 23, 34–36], and did not appreciate their expertise or their ability to competently manage the disease. This was also reported by practice nurses who felt their ability to be involved in patient screening or management were restricted by the preferences of general practitioners [9, 22]. A lack of clear delineation about the role of PC-Ps in the management of CKD resulted in ambiguities and occasional duplication of tasks such as ordering blood tests [5, 7, 8, 27, 29, 31, 34, 36]. Perceptions held by patients about the roles of different healthcare providers was also reported in two studies [8, 9, 30, 34], with one study suggesting that patients could not always differentiate the role of the PC-P compared to nephrologist, and contributed to patient confusion and suboptimal adherence [8, 34].

#### Perceptions about patients

Barriers related to PC-P perceptions regarding patients were reported in eight studies [5, 8, 10, 28, 30–34, 36]. Low patient adherence to management strategies, particularly lifestyle strategies, were reported as a common barrier [5, 28, 30–32, 36]. In addition, PC-P felt that due to the asymptomatic nature of CKD, patients did not understand the seriousness of CKD and were unlikely to prioritise its management until the disease reached a more severe stage with symptoms [8, 10, 30, 33, 34].

#### Other barriers

Barriers relating to ‘beliefs about capabilities’ were reported in five studies [8, 10, 27, 33–35]. Within this domain, the challenging nature of managing CKD within the primary care setting was highlighted [8, 10, 27, 34], especially due to the nature of CKD as an incurable condition with multiple co-morbidities. In addition, educating patients about the management of CKD was reported to be difficult, in part due to limited health literacy of patients [33, 35]. Within the domain of ‘social influences’, insufficient communication between members of the healthcare team was identified as a barrier to the management of CKD in six studies [5, 8, 27, 30, 34–37]. PC-Ps felt that communication between primary care and nephrology services was poor, and there was a general lack of communication back to primary care.

Overall, the most common barrier described in 13 studies was a lack of time [6, 8, 9, 22, 26–35], followed by 10 studies citing a fear by PC-Ps of frightening patients with a diagnosis of CKD [6, 7, 9, 10, 22, 24, 26, 32, 33, 35], and 10 studies which referred to a dissatisfaction with CKD guidelines [6, 8, 10, 24, 25, 27, 28, 31, 32, 34, 35].

### Enablers to identification and management of CKD in primary care

Enablers to the detection and management of CKD in primary care identified in this review were categorised into six domains of the Theoretical Domains Framework. The domains and corresponding themes are shown by study in Table 5, with exemplar quotes for each theme listed in Table 6.

**Table 5:** Enablers to diagnosis and management of CKD in primary care, as reported in included studies (studies listed by reference number)

	[22]	[7]	[32]	[29]	[30]	[23]	[8, 34]	[33]	[24]	[5, 36]	[25]	[31]	[6]	[9]	[26]	[27]	[37]	[10]	[35]	[28]
Beliefs about capabilities																				
Managing patient expectations during education										X						X				
Relationship between primary care provider and patient												X		X		X				
Beliefs about consequences																				
Prioritising patient quality of life																X			X	
Environmental context and resources																				
Access to laboratories												X								
Access to nephrology							X						X			X	X			
Better access to support services																	X			
Financial incentives	X				X		X							X						
Nephrology referral pathways			X							X					X					
Patient education resources							X	X							X	X				
Raising patient awareness of services available					X															
Technological improvements to identify and manage CKD							X	X	X	X	X	X			X					
Time/workload														X						
Knowledge																				
Diagnosis of CKD supports a proactive approach to care		X												X						
Guidelines provide roadmap for care			X				X					X	X			X	X		X	
Training and education							X			X										
Social influences																				
Collaboration between members of health care team							X			X					X	X	X			
Social/professional role and identity																				
Capitalising on credibility from existing services					X															
Clear delineation of healthcare provider roles					X					X		X

**Table 6:** Exemplar quotes illustrating enablers to diagnosis and management of CKD in primary care, by theme^a^

Domain/theme	Quote
Beliefs about capabilities	
Managing patient expectations during education	“I think if you try to set the expectations fairly quickly, then you know that certainly helps.” [27]
Relationship between primary care provider and patient	“Sometimes…if you just gave them time, if you just show them that you really, really care, they go to all the quality of the physician and nurses, then they start to trust you, then they actually start to actually listen to what you’re saying, and then we can have good discussions. So a lot of people will sort of turn around the initial ‘no, I want this, this, this, and that.” [27]
Beliefs about consequences	
Prioritising patient quality of life	“It would have just been a burden to send [the patient] to another specialist, and explain all the story and inevitably the [nephrologist] says “oh let’s do a couple of extra investigations”…for some of these older people, it’s a marathon process.” [27]
Environmental context and resources	
Access to laboratories	“We used to have a lab in our family practice unit, right in the same building and that really was helpful for our patients in terms of any sort of laboratory investigations, but yeah.” [31]
Access to nephrology	“it would make more sense for me as a non-palliative care doctor to be able to quickly access with a phone call somebody who has that information in their head right away” [27] “… if I’m really worried about something, I text the nephrologist I know real well and say…this is what’s going on, it’s in the record, and they get in” [8, 34]
Better access to support services	“Home care service in [a small population center] is very poor. .. they say that are too busy to provide additional services for seniors. Often patients end up in the [emergency room] ER and/or hospital when early intervention could prevent this. Palliative care in this region is also poor. I have taken it on myself to do home visits, etc. to help people at home as long as the patient and family are comfortable.” [37]
Financial incentives	“There had to be some sort of remuneration…so it makes it [CKD service] worth the time…. At the end of the day, we have to run a business and pay for staff so to be able to prioritize time for those different jobs you need to have some sort of income for it” [30]
Nephrology referral pathways	“The Nephrology Department can see the referrals coming in, so they can see how providers, in general, [treat] kidney disease. Are we reasonable with our referrals?...Are we sending people too early or too late? It would be nice to know are there places where there’s room for improvement. I want to know whether I’m doing a reasonable job or not” [26]
Patient education resources	“I had a nice little, laminated handout that came from Nephrology on guidelines and referrals. It has now gone missing, so it would be helpful to have that resent out again – it’s a very convenient and worthwhile thing to have” [26] “I’d love to see a promotion about the kidney class, so that clinicians are more aware of it. . . . if they’d promote the kidney class and say, in general these classes are offered at [these] various times and locations, etc. -. that would help primary care, because we inevitably get those types of questions” [26]
Raising patient awareness of services available	“It’s probably the fact that we don’t have ads on the radio saying go into pharmacy for this and the other…that’s the thing that makes people realize what your scope of business is” [30]
Technological improvements to identify and manage CKD	Automatic alerts (eg BP mgmt) would: “prompt people, even if they’re not fully educated about CKD, to make sure that they do a pretty comprehensive job of managing the disease.” [25]
Technological improvements to identify and manage CKD	“I think for me the most important thing would be just having a shared EMR where you can just look up that encounter very quickly” [8, 34] “I thought it was great [to have it automatically reported], because I didn’t have to try to manually calculate it. Prior I had been using kind of just ballpark numbers to try to guesstimate when I thought somebody’s renal function was starting to decline and if I needed to adjust medication. So, it was challenging because it added work to my day to have to manually do that or try to assess that.. . So it has made life easier for me to have it calculated” [26] “I think it’s a good tool. So the fewer steps that we have to do to get to the right answer, and the right thing to do, the better it is. I think the automatic calculator is quicker and better at math than I am, and more reliable. And so, it takes away some of the potential for error that I might have introduced by manually doing the calculations myself” [26]
Time/workload	“more time to discuss these issues with patients than the general practitioner and can listen and engage [with] the patient” [9]
Knowledge	
Diagnosis of CKD supports a proactive approach to care	“... then you realise they also have CKD so it gives you the level of awareness. This patient has got ... is up the CKD spectrum and we need to be especially aware of how we intervene with their other morbidities.” [7]
Guidelines provide roadmap for care	“just to get that learning out there and to have a readily available tool to go “okay, for this symptom I’ll do this and for these symptoms I’ll do that,” it would be helpful.” [27] “I don’t think [guidelines] should determine [behaviour], but they should definitely guide it and direct it because it’s, again, research based and trying to follow that.” [31] “It was shown very clear when to refer, when you’ve got proteinuria when to refer, when, so that not everyone with proteinuria had to be referred and so the guidelines I thought were very clear and good.” [6]
Social influences	
Collaboration between members of health care team	“It usually involves a multiple health professional team as well as the patient and their family. It rarely is just a patient–physician relationship.” [27] “I just want to be able to call someone for advice and not feel like I am wasting their time. I want a nephrologist to want to help me because I am in the trenches.” [37] “Shared care is essential especially given the workload of these patients. Not ‘my’ patient and not ‘your patient’. Our patient!” [37]
Social/professional role and identity	
Capitalising on credibility from existing services	“What I did is your kidney study was there and then diabetes study we started in the pharmacy, so we linked both together and that has been better. So the same person, we can sometimes do both studies.” [30]
Clear delineation of healthcare provider roles	“I can just send tasks to certain nurses or support staff just to follow back up with them and ask them to order whatever I need to be done.” [31] “As soon as we started teaching the staff members, you know make sure that you ask them this, then it became a lot easier” [30]

^a^In addition to the themes listed in the table, the following theme was identified in the primary studies without quotes provided: *training and education*

#### Environmental context and resources

The most commonly reported enablers to the diagnosis and management of CKD in primary care were related to ‘Environmental context and resources’ and reported in 14 studies [5, 6, 8, 9, 22, 24–27, 30–34, 36, 37]. PC-Ps reported that technological improvements assisted them to identify and manage CKD [5, 8, 24–26, 31, 33, 34, 36]. In particular, shared electronic medical records facilitated collaboration between different healthcare providers, and software programs that automatically calculated eGFR were highlighted as being valuable. PC-Ps described having adequate access to specialists, including for their own professional support, as being highly valuable [6, 8, 27, 34, 37]. The availability of patient education resources [8, 26, 27, 33, 34], and funding for screening and management initiatives [8, 9, 22, 30, 34] were also considered to be enablers to effectively diagnosing and managing patients. Additional enablers included the presence of clear referral pathways to specialist care [5, 26, 32, 36], including guidelines of when to refer; improved access to support services, particularly in regional areas [37]. Other enablers included access to laboratories [31]; raising patient awareness of available services [30]; and, amongst practice nurses, the presence of time to listen to and engage with the patient [9].

#### Knowledge

Ten studies reported enablers for the identification and management of CKD in primary care which aligned with the ‘knowledge’ domain [5–9, 27, 31, 32, 34–37]. PC-Ps highlighted that the value of CKD guidelines was in providing direction for patient care, which enabled PC-Ps to then individualise care [6, 8, 27, 31, 32, 34, 35, 37]. When training opportunities and educational resources were available, they were also considered an important enabler to support CKD management in primary care [5, 8, 34, 36]. Finally, the initial diagnosis of CKD was also identified as an enabler in two studies [7, 9], primarily because awareness of the diagnosis of CKD then facilitated the PC-P to develop a proactive treatment plan.

#### Other enablers

Within the ‘social influences’ domain, five studies [5, 8, 26, 27, 34, 36, 37] highlighted the value of collaboration between members of the healthcare team. In particular, collaboration and clear communication between PC-P and nephrology services was emphasised, although the importance of collaboration between all members of the healthcare team as well at the patient and their family was also highlighted. Three studies reported enablers that aligned with ‘social/professional role and identity’ [5, 30, 31, 36]. The importance of clear role delineation between members of the healthcare team was emphasised in three studies [5, 30, 31, 36]. One study [30] reported that there are opportunities to leverage on existing services, such as when developing a CKD risk assessment service in the community pharmacy setting. Within the ‘beliefs about capabilities’ domain, the importance of the relationship between the PC-P and the patient was described in three studies [9, 27, 31]. In particular the trust developed between the patient and PC-P was an important factor that enabled successful management of CKD. In addition, the importance of managing patient expectations about CKD management during education and ensuring they understood their care plan was also described [5, 27, 36]. Finally, within the ‘beliefs about consequences domain’, PC-Ps in two studies [27, 35] highlighted the value of considering the patient ‘as a whole’, including the impact of a CKD diagnosis on their quality of life, and was described as important to decisions about future management.

Overall, the most common enabler identified was the presence of supportive technology to identify and manage CKD, reported in seven studies [5, 8, 24–26, 31, 33, 34, 36], followed by having a collaborative relationship between members of the healthcare team (reported in five studies, [5, 8, 26, 27, 34, 36, 37]).

## Discussion

To the authors’ knowledge, this is the first systematic review to explore both the barriers and enablers reported by PC-P regarding the diagnosis and management of CKD in the primary healthcare setting. Common barriers included a lack of time for screening and management in the primary care setting, fear about increasing patient anxiety by delivering a diagnosis of CKD, and a perception that CKD guidelines were difficult to use, confusing, or changed frequently. Enablers included the presence of supportive technology for identifying and managing CKD, and a collaborative approach between the healthcare team. Given the high prevalence of CKD worldwide, and the important role of primary care in managing this condition, the findings highlight potential opportunities for improving the detection and management of CKD.

Barriers related to the ‘environmental context and resource’ domain were most commonly reported, particularly a perceived lack of time to treat CKD in the primary healthcare setting [6, 8, 9, 22, 26–35]. This finding aligns with previous systematic reviews relating to CKD specifically [11], and cardiometabolic diseases more broadly [38]. The presence of a high workload and limited time availability in primary care have been previously highlighted in the literature [39]. These barriers are likely to present particular challenges in the case of CKD, a disease known to be associated with multiple co-morbidities. Research suggests that of those individuals diagnosed with CKD, the majority have a least one co-morbid condition, and many patients may have multiple conditions [40, 41]. These conditions are often associated with complex management strategies and require referrals to multiple specialists, which substantially increases the workload associated with managing these conditions in primary care [42]. Previous research has also highlighted the challenges associated with applying clinical practice guidelines to patients with multi-morbidity, given that such guidelines are typically designed for the management of an individual condition [43]. Interestingly, the most common enabler to effective identification and management of CKD in primary care identified in the current review was the presence of supportive technology, for example shared electronic medical records and automatic calculation of risk markers. While limited time and the challenges associated with a multi-morbid condition such as CKD are likely to remain present in the primary care setting, these findings suggest that practical strategies around the use of electronic medical records may in part alleviate these issues, and therefore should be explored further.

As with any chronic condition, effective management of CKD is dependent on both the clinical expertise of the practitioner and appropriate self-management behaviours by patients. This is dependent on the patient being informed and knowledgeable about their condition. The present review highlighted a level of anxiety amongst PC-P about when or if to describe a diagnosis of CKD to patients, with PC-Ps subsequently underplaying the severity of the condition [6, 7, 9, 10, 22, 24, 26, 32, 33, 35]. Previous research conducted in patients with CKD further supports this finding. Daker-White et al. [44] interviewed patients with Stage 3 CKD, and found that limited or partial disclosure of the diagnosis of CKD was common, and the diagnosis of CKD frequently downplayed as ‘nothing to worry about’ or ‘nothing serious’. This approach can trivialise the condition, in turn limiting the ability of the patient to self-manage the condition and restricting their ability to make informed choices regarding their treatment [44]. Previous research has also demonstrated that patient understanding of CKD supports improved outcomes [45, 46], suggesting that hesitation to inform patients may result in poorer health outcomes. Barriers related to knowledge of the diagnosis and management of CKD were also identified in the present review, including a dissatisfaction with existing CKD guidelines and a perceived lack of training on CKD management. The application of clinical practice guidelines to multi-morbid patients is particularly challenging [42, 43]. For patients and carers, managing multi-morbidity in CKD has been described as complex, exhausting and challenging [47]. The timing of the research included in the present review should be considered when interpreting these results, with the majority of studies [5, 6, 8–10, 22–25, 27–31, 33–37] published after the release of global [48] and country-specific CKD management guidelines [49–52], suggesting challenges with guidelines persisted after the release of the most recent guidelines. Despite these challenges, guidelines for care were also described as useful in this review. Taken together, these results highlight the value of guidelines, but emphasise the need to ensure management guidelines consider the complexities of the condition. Furthermore, while a tendency to not disclose or provide limited disclosure about a diagnosis may be well-intentioned, it is vital for patients to make informed choices about the management of this chronic condition.

Due to the complex nature of CKD, the management of CKD requires input from a multi-disciplinary team spanning primary and specialist care. Research has suggested that identification of CKD and adherence to guidelines for management of advanced CKD is greater when a nephrologist is involved in patient care [53, 54], with early referral to nephrology associated with favourable patient outcomes [55]. In addition, continuing of care from a PC-P has been associated with improved blood pressure control in patients with CKD [56]. This highlights the importance of shared care which values the input of all members of the healthcare team. However, the current review identified a number of barriers associated with the functioning of this team, relating to issues pertaining to role identify and social influences. Common barriers described were a perception amongst PC-Ps that other healthcare providers, such as nephrologists, underestimated the importance of their role and their relationship with their patients [5, 8, 23, 34–36], as well as a lack of clear delineation of each provider’s role [5, 7, 8, 27, 29, 31, 34, 36]. Challenges pertaining to inadequate communication between members of the healthcare team was also an important barrier identified [5, 8, 27, 30, 34–37]. Poor collaboration between specialist and primary care providers has been reported previously [57], including in those with complex conditions [58], with CKD patients also reporting problems experienced with coordination of care [59]. In order for effective patient management, there is a need for coordinated and collaborative care which spans all members of the healthcare team. Indeed, a collaborative relationship between members of the healthcare team was also identified as one of the most commonly reported enablers to effective CKD diagnosis and management in the current review [5, 8, 26, 27, 34, 36, 37].

Previous research has described effective strategies to improve collaboration and communication between primary care and nephrology. Haley et al. [60] implemented a quality improvement activity using modified tools from the Renal Physicians Association toolkit. These tools were designed for either the primary care clinician or the nephrologist, and included education on topics including CKD identification, communication between healthcare practitioners, and patient education. Provision of these tools was associated with greater identification of CKD, increased referral to nephrology services, increased communication, and development of co-management plans, and greater healthcare provider satisfaction with co-management. These findings highlight how existing resources can be used to enhance the primary care-nephrology relationship. Increasing access to specialists and support services has also been found to be effective. For example, an intervention involving phone access for PC-P to a range of healthcare providers including nurse navigator, community care resource coordinator, and general internal medicine, supplemented by online access to hospital laboratory results [61], allowed PC-Ps to clarify their role and encouraged collaborative care [58]. Importantly, such interventions address the barriers and enablers to CKD identification and management identified here, such as the importance of role clarification and reciprocal communication, enhancing shared care.

The review had several limitations. A number of the studies included had small sample sizes, limiting the generalisability of their findings. Studies came from multiple countries with varied healthcare systems, meaning some of the findings may not be applicable to all countries. While the limited number of studies meant it was not possible to compare findings between countries, it was observed that commonly reported themes were similar between different countries. This was particularly evident in the case of barriers, although the enabler ‘presence of supportive technology’ appeared to be predominantly observed in studies conducted in the United States and Canada. This finding suggests the presence of common challenges facing primary health practitioners in a number of countries, although potential differences in enablers between countries require further investigation. Similarly, all included studies were limited to published data which focused specifically on primary healthcare, meaning some relevant studies may have not have been detected. All included studies were in the English language, which may have also resulted in the exclusion of potentially relevant articles.

## Conclusion

This systematic review identified a number of barriers and enablers which PC-P face when identifying and managing CKD. Themes relating to ‘environmental context and resources’, ‘beliefs about consequences’ and ‘knowledge’ were the most commonly reported barriers, specifically a lack of time, anxiety communicating a diagnosis of CKD, and a dissatisfaction with current with CKD management guidelines. The presence of supportive technology within practices was the most commonly described enabler, followed by a collaborative relationship between members of the primary healthcare and nephrology team. The findings of this review suggest a need for time-efficient strategies that promote collaboration between members of the healthcare team, and practice guidelines which consider the frequently co-morbid nature of CKD. Enhanced collaboration between PC-P and nephrology services may also support PC-Ps when diagnosing CKD in primary care, and facilitate improved patient self-management.

## Supplementary information

**Additional file 1:** Supplementary Material 1: PRISMA 2009 Checklist. Supplementary Material 2: Example search strategy.

## Data Availability

The datasets used and/or analysed during the current study are available from the corresponding author on reasonable request.

## Abbreviations

CKD: Chronic kidney disease
PC-P: Primary care practitioners
